# Prevalence, associated factors and antimicrobial susceptibility patterns of *Salmonella* and *Shigella* species among diarrheic under five children in Sultan Sheik Hassan Yabere referral Hospital, Jigjiga, Eastern Ethiopia

**DOI:** 10.1186/s12887-024-04755-6

**Published:** 2024-05-06

**Authors:** Kader Muse, Kedir Urgessa, Tadesse Shume, Bawlah Tahir, Fitsum Weldegebreal

**Affiliations:** 1 College of Medicine and Health Science, Department of Medical Laboratory Sciences, Jigjiga University, Jigjiga, Ethiopia; 2 School of Medical Laboratory Sciences, College of Health and Medical Sciences, Haramaya University, Harar, Ethiopia; 3 Laboratory Bacteriology Research, Faculty of Medicine and Health Sciences, Ghent University, De Pintelaan, Ghent, Belgium

**Keywords:** Prevalence, *Salmonella*, *Shigella*, Diarrhea, Drug resistance, Under-five children

## Abstract

**Background:**

Diarrhea caused by *Salmonella* and *Shigella* species are the leading cause of illness especially in developing countries. These infections are considered as the main public health problems in children, including Ethiopia. This study aimed to assess the prevalence, associated factors, and antimicrobial susceptibility patterns of *Salmonella* and *Shigella* species in Sheik Hassan Yabere Referral Hospital Jigjiga, Eastern Ethiopia from August 05 to November 15, 2022.

**Method:**

A cross-sectional study was conducted among 239 under-five children with diarrhea selected through a convenient sampling technique. A structured questionnaire was used to collect associated factors. A stool sample was collected and processed for the identification of *Salmonella* and *Shigella* species using MacConkey adar, Xylose Lysine Deoxycholate agar (Oxoid Ltd) and Biochemical tests. The antimicrobial susceptibility pattern of isolates was performed using the Kirby-Bauer disc diffusion technique. The data was entered into Epi-data version 4.6 and exported to the statistical package of social science version 22 for analysis. The association between outcome and independent variables was assessed using bivariate, multivariable, and chi-square and P-value < 0.05 was considered as statistical significance.

**Result:**

Overall prevalence of *Salmonella* and *Shigella* species was 6.3% (95% CI, 5.7–6.9%), of which 3.8% (95 CI, 3.2–4.4%) were *Salmonella* species and 2.5% (95% CI, 1.95-3%) were *Shigella* species. Unimproved water source (AOR = 5.08, 95% CI = 1.45, 17.25), open field (AOR = 2.3, 95% CI = 1.3, 5.03), rural residence (AOR = 1.8, 95% CI = 1.4, 7.5), Hand-washing practice (*p* = 0.001), and raw meat consumption (*p* = 0.002) were associated with occurrence of *Salmonella* and *Shigella* species. *Salmonella* and *Shigella* isolates were resistant to Ampicilin (100%). However, *Salmonella* isolates was sensitive to Norfloxacin (100%). About 22.2% and 16.7% of *Salmonella* and *Shigella* isolates were multi-drug resistant, respectively.

**Conclusion:**

Prevalence of *Salmonella* and *Shigella* species were lower than most studies done in Ethiopia. Hand-washing habit, water source type, Open field waste disposal habit, raw meat consumption and rural residence were associated with Salmonellosis and shigellosis. All isolated Salmonella were sensitive to norfloxacin. The evidence from this study underscores the need for improved water, sanitation and hygiene (WASH) system and the imperative to implement drug susceptibility tests for the treatment of *Salmonella* and *Shigella* infection.

## Introduction

Diarrheal diseases are common public health problems in many parts of the world including Ethiopia. Globally 21% of deaths in children under the age of 5 years results from diarrheal infection mortality, which translates to 2.5 million child deaths. Africa and South Asia are still home to more than 80% of child deaths [1], making diarrhea the second leading cause of death among children under the age of five [2]. *Salmonella* and *Shigella* are the major cause of childhood diarrhea associated with a high burden of illness n in the developing world where there is poor sanitation and limited improved health care services [3].

In Ethiopia, Diarrheal diseases are the cornerstone in contribution of child mortality; the total prevalence of diarrhea among under five children was (12%) according to the report of Ethiopian demographic Health survey [4], Particularly by *Salmonella* and *Shigella* species, accounting for approximately 8% of all deaths among children under age 5 worldwide [5].

Antibiotic resistance is becoming a significant health problem and leads to a serious threat in public health worldwide. Particularly, Multi-drug resistance pathogen is nowadays increasing which in turn, leads to variety of challenges toward diarrheal disease treatment and control globally [6].

There is no published data on the prevalence, antimicrobial susceptibility and associated factors of *Shigella* and *Salmonella* spp among under-five children in the study area. Therefore, the current study was conducted to determine the prevalence, associated factors and antimicrobial susceptibility patterns of *Salmonella* and *Shigella* species among diarrheic under-five children in Sultan Sheik Hassan Yabere Referral Hospital, Jigjiga, Eastern Ethiopia.

## Materials and methods

### Study area and period

The study was conducted in Jigjiga town at Jigjiga University Sultan Sheik Hassan Yabere Referral Hospital from 05 August to 15 November 2022. Jigjiga town is the capital city of the Somali regional state, 630Km away from Addis-Ababa. Based on the 2007 Census conducted by the Central Statistical Agency of Ethiopia, this city has a total population of 277,560, of whom 149,292 are men and 128,268 women [7]. Jigjiga city has three governmental hospitals where two of them are general hospitals and one of them is specialized referral hospital. Jigjiga University Sheik Hassan Yabare referral hospital is comprehensive specialized referral [8].

### Study design and population

An institutional based cross-sectional study design was used. All under-five children visited Sheik Hassan Yabere Referral Hospital during the study period were source population. All under-five children with diarrhea disease symptoms attended Sheik Hassan Yabere Referral Hospital during study period were the study population. All under-five children passing loose or watery diarrhea three or more times a day who attend the hospital during the study period were included. Children those who were critically ill and unable to give stool sample and those that were on antibiotic treatment with in the last two weeks were excluded.

### Sample size determination and sampling techniques

The sample size was calculated using a single population proportion formula based on the assumption of 5% expected margins of error, considering 95% confidence interval, 10% non-response rate and by taking the prevalence of 17% of *Shigella* spp from previous study conducted in Arbaminch, southern Ethiopia [9]. The final total study subjects of this study were 239. Eligible study participants (children attending under five outpatients) were enrolled sequentially using convenient sampling technique.

### Data and sample collection methods

The data were collected by two professional nurses and two trained medical laboratory professionals. Data on Socio-demographic, socio-economic characteristics, environmental factors and behavioral factors were collected by interview guided pretested structured questionnaire adopted from previous studies [10, 11]. The guardians were instructed to bring a freshly passed diarrhea stool of the study subjects in a sterile stool cup using clean applicator stick and to avoid contamination with urine and other materials. One teaspoonful of diarrhea stool sample was collected using a pre-labeled, clean and sterile screw cup container. The collected diarrhea stool was placed immediately in Cary Blair transport (Oxoid, UK) and transported to Jigjiga University Sultan Sheik Hassan referral hospital microbiology laboratory within 2 h of collection for investigation.

### Bacteriological analysis

Prior to subjecting the sample to culture media, physical analysis of the stool sample was performed in order to check whether the sample was diarrheal or not, presence of blood, pus and mucus. Children with diarrhea, blood, mucus were included. Diarrhea stool specimen was inoculated using sterile wire loop on MacConkey Agar (Oxoid, Ltd), and Xylose lysine- Deoxycholate agar (XLD) (Oxoid Ltd). The inoculated plates ware incubated at 37 °C for 24 h (SOPs) [12]. After 24 h incubation, the plates were checked for colony characteristics of *Salmonella* and *Shigella* species. Furthermore, colorless to yellow colonies on MacConkey agar and pink to red colonies Xylose Lysine Deoxycholate (XLD) (Oxoid Ltd) [12] agar were typically considered as Non-lactose fomenters. In addition, the colonies considered as non-lactose fomenters were further identified by biochemical tests to confirm the identification to genus level of the pathogens Biochemical tests such as klingler iron agar (KIA), motility indole, lysine medium, simon’s citrate agar, and unease test were used [13].

### Antimicrobial susceptibility testing

The drug susceptibility test was done by a disk diffusion method by using Muller Hinton Agar (MHA) according the guidelines of the Clinical Laboratory Standard Institute [14]. Colony suspension was made using normal saline (0.85% NaCl) equivalent to 0.5% McFarland standard from grown overnight colonies [15]. A sterile cotton swab was dipped into the suspension in order to remove additional suspensions by gentle rotation of the swab against the surface of the tube and then spread evenly over the Muller Hinton agar (Oxoid, Ltd). The drugs were selected in accordance with the Ethiopian Drug Administration standard treatment guidelines and Control Authority’s and the Clinical Laboratory Standards Institute’s (CLSI). Based on the drugs those professionals use to treat bacteria diarrheal disease, each and every single of the isolates were tested for ampicilin (10 µg), tetracycline (30 µg), chloramphenicol (10 µg), norfloxacin, (5 µg), ciprofloxacin (5 µg), and ceftriaxone (30 µg) on the Muller Hinton agar, the plate were then incubated at 37^o^C for 18 h and interpreted according Clinical Laboratory Standard Institute [14]. Finally, the diameter of the zone of inhibition was measured and interpreted as sensitive, intermediate, and resistant based on the Clinical and Laboratory Standards Institute [14].

### Data quality control

Questionnaires was primarily written in English, translated to Somali and returned back to English to keep uniformity. Manufacturer instructions were followed during culture media preparation and sterilization. Sterility of culture media was checked by incubating 5% of each batch at 37^o^C overnight and observed for growth. Culture media which show any growth were discarded and replaced by new batch. In addition, Quality control strains of the American Type Culture Collection (ATCC) like *Escherichia* coli ATCC 25,922, *Shigella* dysentery ATCC 13,313 and *Salmonella* typhi ATCC 13,311 [16] were used to check the performance of culture media, biochemical tests and antimicrobial susceptibility testing.

### Method of data analysis

The laboratory analysis result was registered on the laboratory data collection pre-coded format sheet. Data were entered to Epi-data version 4.6 and was analyzed using SPSS version 22. Descriptive statistics and frequency table were used to summarize the data. Chi-square, bivariate and multi-variable analysis were used to predict the association between the dependent and independent variables. Variables with p-value ≤ 0.25 on bivariate logistic regression analysis were selected for multi-variable analysis. Adjusted odds ratio (AOR) with 95% confidence interval (CI) was used to measure the strength of the association. A P-value ≤ 0.05 was considered statistical significance.

### Operational definitions

#### Diarrhea

is the passage of three or more loose or liquid stools per day [17].

**Multi Drug Resistant (MDR)** is a antimicrobial resistance shown by microorganism to at least one antimicrobial drug in three or more antimicrobial categories [18].

**Improved drinking water** is a source that, by the nature of construction, adequately protects from outside contamination, include piped water into a dwelling, yard of plot, public tap or standpipe, tube-well or borehole, protected spring, protected dug well, and rainwater collection [19].

**Unimproved sources of drinking water** include unprotected well (dug); unprotected spring, cart with small tank or drum; tanker truck-provided water, surface water [19].

## Results

### Socio-demographic characteristics, behavioral and environmental characteristics

A total of 239 patients with diarrhea were included in this study, with a response rate of 100%. About half 121/239 (50.6%) of the children were male. The mean age (± SD) of the study participants was 2.1(± 0.8) years with the age of range of 0–5 years. Most (83.7%) of the study participants were from urban area and 72.4% of the parents/guardians were illiterate in educational status (Table 1**).**

**Table 1:** Socio-demographic characteristics of the parent/guardian and participants attended at Sheik Hassan Yabere Referral Hospital Eastern Ethiopia, 2022 (*n* = 239)

Variable	Category	Frequency	Percentile (%)
Sex of the child	Female	118	49.4%
	Male	121	50.6%
Age in year	< 2	148	61.9%
	2–5	91	38.1%
Residence	Rural	39	16.3%
	Urban	200	83.7%
Educational status of the parents/guardians	Did not attend school at all (Illetirate)	173	72.4%
	Able to read and write (Literate)	66	27.6%

Majority (73.2%) of the study participants wash their hands after toilet usage. About 70.7% of the study participants utilized improved water sources and 71.5% of the parents /care givers used waste bin for waste disposal (Table 2).

**Table 2:** Behavioral and environmental characteristics of parent/guardian and patients with diarrhea attending at Sheik Hassan Yabere Referral Hospital jigjiga town, Eastern Ethiopia, 2022 (*n* = 239)

Variable	Category	Frequency	Percentage (%)
Hand washing status the parents/guardians	No	64	26.8%
	Yes	175	73.2%
Raw meat consumption of the child	Yes	14	5.9%
	No	225	94.1%
Finger nail status of the child	untrimmed	62	25.1%
	Trimmed	177	74.9%
Tissue paper usage	No	84	35.1%
	Yes	155	64.9%
Water sources	Unimproved	70	29.3%
	Improved	169	70.7%
Waste disposal system	Open field	68	28.5%
	Waste pin and disposed	171	71.5%

### Prevalence of *Salmonella* and *Shigella* spp

The overall prevalence of isolated *Salmonella* and *Shigella* species were 6.3% (15/239) (95% CI; 5.7-6.9%). Among them, more than half of the cases (3.8%, 95%CI; 3.2–4.4%) individuals were *Salmonella* spp. whereas, the remaining cases (2.5%, 95% CI; 1.95-3%)) patients were *Shigell*a spp. The magnitude of *Salmonella* and *Shigella* spp were higher in parents/guardians had no hand washing practice (18.8%) followed by parents with raw meat consumption (14.3%) and used unimproved water sources (14.3%) (Table 3).

### Factors associated with *Salmonella* and *Shigella* infection

Association between some of the potential factors and prevalence of bacterial diarrhea were assessed using logistic regression analysis. In bivariate logistic regression analysis; sex, residence, water source and waste disposal system were found to be significant (*p* < 0.25) and were considered as a candidate for multivariable logistic regression analysis. In multivariable, logistic regression analysis, water source, waste disposal system and residence found to be statistically significant with positivity rate of *Salmonella* and *Shigella* isolates at *p* < 0.05.

Water sources type (AOR = 5.08, 95% CI = 1.497–17.251), Open field waste disposal habit (AOR = 2.3, 95% CI = 1.3, 5.03), rural residence (AOR = 1.8, 95% CI = 1.4, 7.5) were associated with *Salmonella* and *Shigella* infections (Table 3). Moreover, variables those did not fulfill the assumption of logistic regression analysis were assessed chi-square test such hand-washing habit, raw meat conception and educational status. In chi-square test hand-washing habit and raw meat consumption were found to be statistically significant. Children from parents/guards who had not habit of washing their hands were (*p* = 0.001) more likely to be infected with *Salmonella* spp and *Shigella* spp compared with children from parents/guards who had habit of hand washing. Children who were eating raw meat were (*p* = 0.002) more likely to be infected with *Salmonella* spp and *Shigella* spp compared to these who don’t eat (Table 4).

**Table 3:** Bivariate and Multivariable analysis of factors associated with *Salmonella* and *Shigella* spp among diarrheic under-five children attending at Sultan Sheik Hassan Yabere Referral Hospital, Eastern Ethiopia, 2022

Variable	Category	Culture result of *Salmonella*/*Shigella* isolates		COR (95%CI)	P-value	AOR (95%CI)	P- value
		*Yes*	No				
Sex	Female	5 (4.2%)	113 (95.8%)	0.49 (0.16–1.5)	0.21	0.51(0.15–1.8)	0.3
	Male	10 (8.3%)	111(91.7%)	1(ref)		1(ref)	
Age in year	< 2	9(6.1%)	139 (93.9%)	0.92(0.32–2.7)	0.87		
	2–5	6(6.6%)	85 (93.4%)	1(ref)			
Residence	Rural	5 (12.8%)	34 (87.2%)	2.8(0.899–8.683)	0.08	1.8(1.4–7.5)	**0.004**
	Urban	10 (5%)	190 (95%)	1(ref)		1(ref)	
Finger nail status	Untrimmed	5(8.1%)	57(91.9%)	1.46(0.48–4.67)	0.5		
	Trimmed	10(5.6%)	167(94.4%)	1(ref)			
Tissue paper usage	No	7(8.3%)	77(91.7%)	1.67(0.584–4.779)	0.3		
	Yes	8(5.2%)	147(94.8%)	1(ref)			
Water source	unimproved	10(14.3%)	60(85.7%)	5.46(1.795–16.6)	0.003	5.08(1.45–17.25)	**0.001**
	Improved	5(3%)	164(97%)	1(ref)		1(ref)	
Waste disposal system	Open field	8(11.8)	60(88.2%)	3.1 (1.1–8.9)	0.04*	2.3(1.3–5.03)	**0.02**
	Waste pin &disposed	7(4.1%)	164(95.9%)	1(ref)		1(ref)	

COR: Crude odds ratio, AOR: Adjusted odds ratio, Ref: Reference category, CI: Confidence interval

**Table 4:** Chi-square of associated factors with *Salmonella* and *Shigella* among diarrheic under-five children at Sheik Hassan yabere Referral Hospital Eastern Ethiopia, 2022

Variable	Category	Culture Result *of Salmonella*/*Shigella* isolates		Chi-square	P value
		Yes	*No*		
Educational status of parent/guardians	Did not attend school at all (Illiterate)	11 (6.4%)	162(93.6%)	3.2	1.0
	Able to read and write (Literate)	4(6.1%)	62(93.9%)	1(ref)	
Hand washing practice	No	12(18.7%)	52(81.3%)	20.3	**0.001**
	Yes	3(1.7%)	172(98.3%)	1(ref)	
Raw meat consumption	Yes	2(14.3%)	12(85.7%)	1.8	**0.002**
	No	13(5.8%)	212(94.2%)	1(ref)	

### Antimicrobial susceptibility patterns and multi-drug resistant pattern

To determine antimicrobial susceptibility patterns of isolates, those 15 bacterial isolates were tested for six different antimicrobial discs. All *Salmonella* isolates were sensitive to norfloxacin. On the other hand, all *Salmonella* and *Shigella* isolates showed (100%) resistance rate to ampicillin. In addition, majority *Shigella* spp. isolates were resistant to tetracycline (83.3%). Moreover, one-third of *Salmonella* and *Shigella* spp. isolates were found to be resistant to ciprofloxacin **(**Table 5**).** Furthermore, Two of the nine *Salmonella* isolates, and one of the six *Shigella* isolates were multi-drug resistant to ampicillin, tetracycline and Chloramphenicol.

**Table 5:** Antimicrobial susceptibility patterns of *Salmonella* and *Shigella* isolates from diarrheic under-five children attending Sultan Sheik Hassan Yabere Referral Hospital, Eastern Ethiopia, 2022

Antimicrobial disks	*Salmonella* isolates *n* = 9			*Shigella* isolates *n* = 6		
	S N (%)	I N (%)	R N (%)	S N (%)	I N (%)	R N (%)
Ampicillin (10 µg)	-	-	9(100%)	-	-	6(100)
Ciprofloxacin (5 µg)	6(66.7)	3(33.3%)	-	4(66.7%)	2(33.3%)	
Ceftriaxone(30 µg)	7(77.8%)	1(11.1%)	1(11.1%)	4(66.7%)	-	2(33.3%)
Norfloxacin(10 µg)	9(100%)	-	-	4(66.7%)	2(33.3%)	-
Chloramphenicol (30 µg)	3(33.4%)	3(33.3%)	3(33.3%)	1(16.7%)	2(33.3%)	3(50%)
Tetracycline (30 µg)	2(22.2%)	2(22.2%)	5(55.6%)	-	1(16.7%)	5(83.3%)

## Discussion

*Salmonella* and *Shigella* are common diarrheagenic bacterial pathogens in the worldwide. Both are more predominant in under-five children [20]. This study was determining the prevalence and factors associated with *Salmonella* and *Shigella* spp in diarrheic under-five children. In this study the prevalence of *Salmonella* and *Shigella* spp were (6.3%). And hand-washing habit, raw meat consumption, residence, waste disposal and water source were remained statistically significant at (*p* < 0.05) with diarrhea caused by *Salmonella* and *Shigella* spp.

In this study, the overall prevalence of *Salmonella* and *Shigella* spp were 6.3%. It is similar with study done in Jimma, Ethiopia (6.2%) [21], but it is lower than studies done in Arbaminch (17.45%) [9], and Nekemte Referral Hospital Oromia, Ethiopia (9.2%) [10]. However, the prevalence was higher than that reported in Ambo town, Ethiopia (3.86%) [11]. The discrepancy might be due to a difference in the study time and illness due to other enteric pathogens. And Younger age is highly infected with bacterial diarrheal disease [22].

In the current study, the prevalence of *Salmonella* isolates was 3.8%, which is comparable with studies done in Kenya (3.5%) [23], and Addis Ababa (3.95%), Ethiopia [24]. In contrast our result is higher than the studies conducted in Turkey (1.5%) [25], Nepal (1%) [26], Windhoek, Namibia (2.6%) [27], Hawassa, Southern Ethiopia (2.5%) [28]. However, this finding is lower than studies done in Harar (6.7%) [29], and Dessie (5.2%) Ethiopia [30]. The variation may be due to the difference in the socio-economic status, source of drinking water supply and sanitation. And also, the difference might be due to the divergence of enteric bacterial pathogens since they vary globally from region to region [27].

In the present study, *Shigella* isolates was 2.5% (95% CI, 1.95-3%). This is agreement with studies done in Kenya (2%) [23], and Jimma, Southern Ethiopia (2.5%) [31]. However, our finding is lower than studies done in Nepal (4.6%) [26], Tehran (7%) [32], studies done in Ethiopia; Mekelle, Ethiopia (6.9%) [33], Nigist Eleni Mohammed (8.3%) [34], Addis Ababa (9.1%) [24], Bahirdar (9.5%) [35], Butajira (4.5%) [36], Robe General Hospital (4.3%) [37], Hawassa town (7%) [28], and Harar (11.5%) [29]. The variation could be due to deference in personal hygiene and environmental sanitation and even between and within countries in an identical geographical region.

Hand-washing practice after toilet by parent/guardians was less likely to be infected with the isolates of *Salmonella* and *Shigella* spp. This is in agreement with studies conducted in Igembe district Hospital, Kenya [38], Robe Ethiopia [37], Arbaminch, Ethiopia (2.0, 18.2)] [9]. This could be due to the fact that hand washing is important in reducing occurrence of diarrhea when supported with the availability of water and hand washing facility [39].

In this study, water source was significantly associated with the isolates of *Salmonella* and *Shigella* spp with those who used unimproved drinking water had more likely contracting Salmonellosis and Shigellosis than those who used improved water. This is agreement with study conducted in Dessie, Ethiopia [30], Robe, Ethiopia [37]. It was indicated that the consumption of contaminated food and/ or water is responsible for diarrheal diseases caused by *Salmonella* and *Shigella* isolates [37].

Waste disposal system was significantly associated with the isolates of *Salmonella* and *Shigella* spp with children who live environment with open field disposal system were more likely to be infected by *Salmonella* spp and *Shigella* spp than children from waste and disposed environment. This is compared with the study conducted in Arbaminch, southern Ethiopia [9]. Many disease caused by agents responsible in appropriate waste disposal system have been well characterized [40].

In the current study, consumption of raw meat was significantly associated with the isolates of *Salmonella* and *Shigella* species with those did not eat are less likely contracting by Salmonellosis and Shigellosis than those ate raw meat this is supported with the study done in Gondar, Ethiopia [41], Addis Ababa, Ethiopia [24]. This is supported that raw meat consumption is predictors of diarrheal diseases [42].

In this study, children who were from rural residents were more likely to be infected *Salmonella* and *Shigella* species when compared with those from urban area. This is constant with study done in Harar, Eastern Ethiopia [43]. Because the majority of study participants were being from rural settings, and host fecal flora might be the source of the infection [43].

In this study, *Shigella* isolates was sensitive to norfloxacin (100%). This finding is concordant with the previous study done in Mohamed Memorial Hospital, South Ethiopia (100%) [34] that showed the susceptibility to norfloxacin (100%). However, in this study *Shigella* isolates was resistant to ampicillin (100%), and tetracycline (83.3%). This result is in agreement with study done in, Addis Ababa, Ethiopia (95.7%) [24], and Ambo, Ethiopia (83.5%) [11]. However, the finding of this study report revealed that a relatively low rate of ampicillin-resistant *Shigella* species was isolated compared to the findings reported from Hawassa (63.6%) [28].This difference might be due to miss use of antimicrobial agents without confirmation of etiologic agent.

In this study, *Salmonella* isolates were sensitive to norfloxacin (100%) and ceftriaxone (77.8%). This result is in line with the studies done in Nigist Eleni Mohamed Memorial Hospital, South Ethiopia indicated sensitivity to norfloxacin (100%) [34] and ceftriaxone (77.9%) in Alamura Health center Southern Ethiopia [44]. But in the current study the bacteria was resistant to ampicillin (100%) that is similar with study conducted in Nigist Eleni Mohamed Memorial Hospital, Ethiopia ampicillin (100%) [34]. However, it is slightly different with the studies performed in Addis Ababa, Ethiopia (80%) [24]. The main reason might be frequent use of these antibiotics, different strains and variation in the number of isolates.

The overall prevalence of multi-drug resistance (MDR) among the isolates was 20% which is comparable with study done in rural Mozambique (23%) [45]. But it is lower than studies done in Butajira (47.1%) [36]. Variation might be different in prescription practice among health care providers and different in usage of drugs by the patients.

### Limitations of this study

The current study did not identify bacteria species level due to the lack of anti-sera in the local market. The current study did not identify other cause of diarrhea like virus. In addition, the association between types of diarrhea and educational status of the children with isolated pathogens has not been analyzed.

## Conclusion

The prevalence of *Salmonella* and *Shigella* spp was lower compared with other studies reported in Ethiopia. Under-five children from parents/guardians with poor habit of hand washing practice after toilet, drink water from unimproved sources, consumption of raw meat, and those did not use waste bin and disposed were more likely to be infected with the isolates of *Salmonella* and *Shigella*. All *Salmonella* and *Shigella* spp isolates were sensitive to norfloxacin. This study also revealed that ciprofloxacin can be used as the drug of choice against *Shigella* and *Salmonella* species. Health care workers and stakeholders should focus on water, sanitation and hygiene and improved environmental sanitation to prevent these diseases in pediatric population.

## Data Availability

The data sets generated during and/or analyzed during the current study are available from the corresponding authors on reasonable request.
